# The efficacy of intravenous thrombolysis in acute ischemic stroke patients with white matter hyperintensity

**DOI:** 10.1002/brb3.1149

**Published:** 2018-10-31

**Authors:** Yanyan Liu, Min Zhang, Hanmo Bao, Zhixiang Zhang, Yuqing Mei, Wenwei Yun, Xianju Zhou

**Affiliations:** ^1^ Department of Neurology, Laboratory of Neurological Diseases Changzhou No. 2 People's Hospital, The Affiliated Hospital of Nanjing Medical University Changzhou China; ^2^ The Affiliated Brain Hospital of Nanjing Medical University Nanjing China; ^3^ Emergency Center of the Affiliated Hospital of Xuzhou Medical University Xuzhou China

**Keywords:** acute ischemic stroke, deep white matter hyperintensity, efficacy, intravenous thrombolysis, periventricular hyperintensity

## Abstract

**Objectives:**

We aimed to investigate effects of deep white matter hyperintensity (DWMH) and periventricular hyperintensity (PVH) on the efficacy of intravenous thrombolysis (IVT) in patients with acute ischemic stroke (AIS).

**Methods:**

A total of 113 AIS patients with WMH were categorized into the PVH group and the DWMH group according to the lesion location, with the division of two subgroups based on whether or not they received IVT treatment: the thrombolysis group and the control group. Kaplan–Meier analysis was used for proportional hazards of recurrent stroke. Further, multivariate Cox regression analysis was employed.

**Results:**

Of total patients, there were 62 PVH patients and 51 DWMH patients: 27 of PVH patients and 22 of DWMH patients received IVT, and the remaining patients only received routine treatment. DWMH patients had a higher risk of END (36.4% vs. 11.1%; *p = *0.034) and HT (22.7% vs. 3.7%; *p = *0.038) than PVH patients in the thrombolysis group. Moreover, DWMH patients undergoing IVT also had a higher risk of END (36.4% vs. 10.3%; *x* ^2^ = 5.050; *p = *0.025) and HT (22.7% vs. 3.4%; *x* ^2^ = 4.664; *p = *0.031) than DWMH patients without IVT. Again, PVH patients had a higher rate of recurrent stroke (20.0% vs. 3.4%; *p* = 0.034) than DWMH patients in the control group after 90‐day follow‐up. Kaplan–Meier analysis showed a significant difference in cumulative probability of no major endpoint events (*p* = 0.039). Further, multivariate Cox regression revealed that PVH is an independent risk factor for stroke recurrence in AIS patients after adjusting confounding factors.

**Conclusions:**

The location of WMH is closely associated with the efficacy of IVT in AIS patients.

## 1 INTRODUCTION

White matter hyperintensity (WMH) is frequently viewed as hyperintensity signal on the T2‐weighted or fluid‐attenuated inversion recovery (FLAIR) magnetic resonance imaging (MRI) sequences, which is closely relevant to ischemic stroke (Shim et al., 2015). Many investigators found that WMH is always associated with early neurological deterioration (END) and large infarct size in patients with acute ischemic stroke (AIS) after intravenous thrombolysis (IVT; Feng et al., 2014; Kongbunkiat et al., 2017; Zhong et al., 2016). They also observed that severe WMH is an independent risk factor for hemorrhagic transformation (HT) and poor functional outcomes after IVT in AIS patients (Gilberti et al., 2017; Kongbunkiat et al., 2017). However, its mechanisms and etiologies are still unclear. Previous studies suggested that the pathological mechanisms for WMH might be associated with vascular diseases including endothelial damage, arteriosclerosis, and Virchow–Robin space dilatation (Pantoni, 2010; Zhang et al., 2015). Moreover, other neuropathological mechanisms, such as severe demyelination, decreased oligodendrocytes, and the proliferation of glial cells (Nordahl et al., 2006), were also suggested. With the severity of WMH, self‐regulation dysfunction of microvascular circulation occurs, leading to thickening, stenosis, or occlusion of small arteries, and eventually leading to low reperfusion of brain tissue that transforms into infarction (Shim et al., 2015). These findings also suggest other potential mechanisms such as blood–brain barrier disruption and microglial cell‐related inflammatory processes (Wardlaw, Smith, & Dichgans, 2013).

According to the site, WMH is often classified into deep white matter hyperintensity (DWMH) and periventricular hyperintensity (PVH). At present, it is widely accepted that WMH lesion at different locations has different mechanisms (Fazekas et al., 1993). For example, PVH is associated with neurofibrillary tangles and arteriosclerosis (Bahrani et al., 2017; Gao et al., 2014). Yet, DWMH is relevant to microinfarction and cerebral hemorrhage (Gao et al., 2014; Shim et al., 2015). Based on these data, we hypothesized that the efficacy of IVT in AIS patients with WMH at different sites may be different. Thus, the objective of this study was to evaluate the efficacy of IVT in AIS patients with PVH or DWMH.

## 2 METHODS

### 2.1 Participants

In this study, a total of 113 AIS patients were consecutively enrolled from department of neurology in Changzhou No.2 People's Hospital between January 2013 and April 2017. All patients were categorized into two groups according to the site of WMH. Of these patients, 51 patients were confirmed with DWMH and 62 patients with PVH by the MRI scan. According to the patients whether or not received IVT, they were further divided into two respective subgroups: the thrombolysis group and the control group. The control patients did not receive IVT because they did not meet the requirement of the therapeutic window for IVT or refused IVT or had other contraindications to IVT**.** The inclusion criteria were as follows: (a) first‐ever ischemic stroke diagnosed by the experienced neurological physician based on diffusion‐weighted imaging (DWI); (b) ranged 6–16 in baseline National Institutes of Health Stroke Scale (NIHSS) score; (c) receiving rt‐PA IVT within 4.5‐hr from onset in the thrombolysis group or not receiving IVT within 6‐hr from onset at admission in the control group; (d) underwent MRI and magnetic resonance angiography (MRA) and CT during hospitalization; (e) ranged 2–3 score by the Fazekas scale for PVH or DWMH; and (f) obtained written informed consent form from participants. The exclusion criteria were as follows: (a) treatment with arterial thrombolysis or IVT before admission; (b) a history of multiple infarctions; (c) failure to undergo MRI and MRA or poor imaging quality; and (d) a diagnosis of hemorrhagic infarct. This retrospective study was approved by the Ethics Committee of Changzhou No. 2 People's Hospital.

### 2.2 Treatments

Besides routine treatments for cerebral infarction such as antiplatelet, brain protection, lipid‐lowering, stabilizing plaques, the patients with AIS in the thrombolysis group received the recombinant tissue plasminogen activator (rt‐PA, 0.9 mg/kg up to a maximum of 90 mg). Ten percent of total dosage of rt‐PA was delivered by intravenous injection within one minute, while the remaining was administrated by intravenous drip within 1 hr. The control group only received routine treatment.

### 2.3 Clinical data collection

Clinical data such as demographical data, risk factors for stroke, including hypertension, diabetes mellitus, hyperlipidemia, coronary artery disease, atrial fibrillation, transient ischemic attack (TIA), the history of smoking and drinking, and the time of onset to treatment, were collected. The neurological deficit was assessed by the National Institute of Health Stroke Scale (NIHSS) at admission and 24 hr later; END was defined as an increase of ≥1 point on the motor NIHSS score or ≥2 points on the total NIHSS score 72 hr after admission (Jeong, Kim, Yang, Han, & Bae, 2015). The motor function was scored using Fugl‐Meyer rating scale (FMS) at admission and 90‐day follow‐up (we chose FMS but not mRS based on the following reasons: Cerebral infarction caused by middle cerebral artery occlusion had a greater influence on motor function of limbs; and FMS is more accurate and reliable than mRS score in evaluating limb motor function (Gladstone, Danells, & Black, 2002). During 90‐day follow‐up, hemorrhagic transformation (HT) and symptomatic intracranial hemorrhage (sICH) defined by ECASS II (Larrue, Kummer, Muller, & Bluhmki, 2001), stroke recurrence, and death were recorded. One neurologist carried out the evaluation of the neurological function and limb function prognosis of the enrolled patients and was blinded to the treatment.

### 2.4 Endpoint events during follow‐up

All patients were followed up for 7, 30, 60, and 90 days after discharge. The major endpoint events included recurrent stroke and death; the secondary endpoint events were other vascular events. Stroke recurrence was defined according to a previous reference (Erdur et al., 2015) as a new neurological deficit lasting for more than 24 hr; the new neurological deficit occurred 24 hr after first‐ever event, not due to other systemic or neurological caused, confirmed by cranial MRI.

### 2.5 Imaging protocol

Cerebral MRI and magnetic resonance angiography (MRA) were performed using a 3.0‐T system (Achieva 3.0; Philips, Royal Philips, the Netherlands). At admission, all participants underwent MRI including DWI sequence and FLAIR sequence. We assessed WMHs by using the Fazekas scale for PVH (0–3 score) or for DWMH (0–3 score) on axial FLAIR images. PVH indicated WMHs in contact with the ventricular wall graded from 0 to 3 as described previously (Shrestha et al., 2009). DWMH indicated that WMHs appeared in the deep white matter graded from 0 to 3 as described previously (Gao, et al., 2014) and isolated from the ventricular wall by a strip of normal‐appearing white matter. We included patients with only PVH or only DWMH scored 2–3 points using the Fazekas scale, while patients with the combination of PVH and DWMH were excluded. One neuroradiologist performed the classification of WMH site and was blinded to the treatment.

### 2.6 Statistics analysis

The Statistical Package for the Social Sciences (SPSS) version 20.0 software package was used for statistical analysis. Continuous variables were presented as mean ± standard deviation (*SD*) and analyzed by using independent‐samples two‐tailed *t* test. Categorical variables were shown as proportions or frequency and analyzed by using chi‐square test or Fisher's exact test. The survival rate of patients with DWMH and patients with PVH without major endpoint events was shown by Kaplan–Meier curve. Further, multivariable Cox regression model was used to analyze relevant risk factors for stroke recurrence. Previous studies showed that age, baseline NIHSS score, baseline FMS score, hypertension, diabetes, coronary heart disease, atrial fibrillation, and transient ischemic attack are risk factors for long‐term prognosis of patients with cerebral infarction (Johnston et al., 2000; Kimura et al., 2009; Parsons et al., 2002), and thus, these independent variables were included in the multivariate regression. Additionally, WHM and IVT were the focus of this study and thus were also included in the regression. *p* Value <0.05 was considered statistically significant.

## 3 RESULTS

### 3.1 Characteristics of patients

We eventually recruited 113 AIS patients with a mean age of 67.5 ± 10.9 years into this study. Of total patients, 62 (54.9%) patients exhibited PVH, while 51 (45.1%) patients showed DWMH. In the whole cohort, 19 (16.8%) patients and 10 (8.9%) patients showed END and HT, respectively. Patients with DWMH had a higher rate of occurrence in END and HT as compared to patients with PVH. Characteristics of enrolled patients and comparisons in clinical outcomes between PVH patients and DWMH patients are presented in Table 1.

**Table 1:** Characteristics of enrolled patients and comparisons in clinical outcomes between PVH and DWMH patients

Characteristics	All patients (*n* = 113)	PVH (*n* = 62)	DWMH (*n* = 51)	*t/x* ^2^	*p*
Age (years, mean ± *SD*)	67.5 ± 10.9	68.4 ± 10.5	66.4 ± 11.4	0.770	0.382
Male, *N* (%)	67 (59.3)	34 (54.8)	33 (64.7%)	1.129	0.288
Baseline NIHSS score (mean ± *SD*)	9.3 ± 3.5	9.4 ± 3.7	9.2 ± 3.5	0.844	0.360
Baseline FMS score (mean ± *SD*)	85.3 ± 8.5	84.9 ± 8.7	85.8 ± 8.1	1.229	0.270
Risk factors					
Hypertension, *N* (%)	91 (80.5)	44 (71.0)	47 (92.2)	8.013	**0.005**
Diabetes mellitus, *N* (%)	53 (46.9)	28 (45.2)	25 (49.0)	0.167	0.683
Hyperlipidemia, *N* (%)	31 (27.4)	17 (27.4)	14 (27.5)	0.000	0.997
Coronary artery disease, *N* (%)	12 (10.6)	6 (9.7)	6 (11.8)	0.128	0.720
Arterial fibrillation, *N* (%)	12 (10.6)	7 (11.3)	5 (9.8)	0.065	0.799
TIA, *N* (%)	11 (9.7)	5 (8.1)	6 (11.8)	0.434	0.510
Smoking, *N* (%)	36 (31.9)	19 (30.6)	17 (33.3)	0.093	0.760
Drinking, *N* (%)	19 (16.8)	12 (19.4)	7 (13.7)	0.634	0.426
Clinical variables					
END, *N* (%)	19 (16.8)	8 (12.9)	11 (21.6)	1.502	0.220
HT, *N* (%)	10 (8.9)	4 (6.5)	6 (11.8)	0.979	0.322
sICH, *N* (%)	4 (3.5)	2 (3.2)	2 (3.9)	0.039	0.842
Recurrence of stroke, *N* (%)	13 (11.5)	11 (17.7)	2 (3.9)	5.250	**0.022**
90‐day death, *N* (%)	5 (4.4)	2 (3.2)	3 (5.9)	0.465	0.495
90‐day FMS score (mean ± *SD*)	86.6 ± 21.2	83.1 ± 24.8	90.9 ± 14.7	7.921	**0.006**
Thrombolysis selection					
Thrombolysis, *N* (%)	49 (43.4)	27 (43.5)	22 (43.1)	0.002	0.965
No thrombolysis, *N* (%)	64 (56.6)	35 (56.5)	29 (56.9)		

Note

Statistical significances are indicated in bold.

DWMH: deep white matter hyperintensity; END: early neurological deterioration; FMS: Fugl‐Meyer rating scale; HT: hemorrhagic transformation; NIHSS: National Institute of Health Stroke Scale; PVH: periventricular hyperintensity; *SD*: standard deviation; sICH: symptomatic intracranial hemorrhage; TIA: transient ischemic attack.

### 3.2 Comparisons in clinical information and clinical outcomes between PVH patients and DWMH patients in the thrombolysis group or in the control group

Among PVH patients, 27 patients (43.5%) received IVT, while 35 patients (56.5%) failed to receive IVT due to beyond therapeutic window (*n* = 25), refusing IVT (*n* = 8), or contraindications (*n* = 2). Of 51 DWMH patients, 22 patients (43.1%) received IVT, and 29 patients (56.9%) failed to receive IVT due to beyond therapeutic window (*n* = 17), refusing IVT (*n* = 9), or contraindications (*n* = 3; Table 2). By comparison, there were no significant differences in age, sex, diabetes mellitus, hyperlipidemia, coronary artery disease, arterial fibrillation, TIA, smoking, drinking, baseline NIHSS score, 24‐hr NIHSS score, baseline FMS score, and 90‐day FMS score between PVH patients and DWMH patients in the thrombolysis group or in the control group (Table 2). But DWMH patients had a higher occurrence of hypertension than PVH patients in the thrombolysis group and in the control group (Table 2). Furthermore, DWMH patients had a higher risk of END and HT than PVH patients in the thrombolysis group but not in the control group (Table 2). In contrast, PVH patients had a higher risk of recurrent stroke than DWMH patients in the control group, but not in the thrombolysis group (Table 2). However, there were no significant differences in sICH and 90‐day death between PVH patients and DWMH patients in the two subgroups (Table 2). Therefore, these data suggested that the efficacy of intravenous thrombolysis is different in AIS patients with WMH at different sites.

**Table 2:** Baseline data and clinical features of patients with white matter hyperintensity at different sites

Characteristics	Thrombolysis		*t/x* ^2^	*p*	Control		*t/x* ^2^	*p*
	PVH (*n* = 27)	DWMH (*n* = 22)			PVH (*n* = 35)	DWMH (*n* = 29)		
Age (mean ± *SD*)	68.0 ± 12.3	63.2 ± 12.7	1.334^a^	0.189	68.8 ± 8.9	68.8 ± 10.0	0.018^a^	0.986
Male, *N* (%)	18 (66.7)	16 (72.7)	0.210	0.647	16 (45.7)	17 (58.6)	1.058	0.304
Risk factors								
Hypertension, *N* (%)	18 (66.7)	20 (90.9)	4.412	**0.036**	26 (74.3)	27 (93.1)	4.274	**0.039**
Diabetes mellitus, *N* (%)	9 (33.3)	9 (40.9)	0.299	0.584	19 (54.3)	16 (55.2)	0.005	0.943
Hyperlipidemia, *N* (%)	7 (25.9)	5 (22.7)	0.067	0.796	10 (28.6)	9 (31.0)	0.046	0.830
Coronary artery disease, *N* (%)	3 (11.1)	2 (9.1)	0.054	0.816	3 (8.6)	4 (13.8)	0.442	0.506
Arterial fibrillation, *N* (%)	3 (11.1)	2 (9.1)	0.054	0.816	4 (11.4)	3 (10.3)	0.019	0.890
TIA, *N* (%)	2 (7.4)	4 (18.2)	1.313	0.252	3 (8.6)	2 (6.9)	0.062	0.803
Smoking, *N* (%)	10 (37.0)	9 (40.9)	0.077	0.782	9 (25.7)	8 (27.6)	0.028	0.866
Drinking, *N* (%)	6 (22.1)	4 (18.2)	0.123	0.726	6 (17.1)	3 (10.3)	0.620	0.431
Baseline NIHSS score (mean ± *SD*)	10.2 ± 4.0	9.0 ± 3.8	1.130^a^	0.264	8.8 ± 3.3	8.9 ± 2.8	0.132^a^	0.895
24‐hr NIHSS score (mean ± *SD*)	8.2 ± 7.0	7.9 ± 5.4	0.155^a^	0.877	8.8 ± 3.7	9.0 ± 4.0	0.272^a^	0.787
Baseline FMS score (mean ± *SD*)	83.0 ± 9.1	86.2 ± 8.7	1.262^a^	0.213	86.4 ± 8.3	85.5 ± 8.0	0.464^a^	0.644
90‐day FMS score (mean ± *SD*)	80.9 ± 26.1	89.7 ± 21.3	1.284^a^	0.205	84.8 ± 24.3	91.7 ± 7.4	1.604^a^	0.116
Clinical variables								
END, *N* (%)	3 (11.1)	8 (36.4)	4.510	**0.034**	5 (14.3)	3 (10.3)	0.228	0.633
HT, *N* (%)	1 (3.7)	5 (22.7)	4.298	**0.038**	3 (8.6)	1 (3.4)	0.750	0.386
sICH, *N* (%)	1 (3.7)	1 (4.5)	0.022	0.883	1 (2.9)	1 (3.4)	0.018	0.893
Recurrent stroke, *N* (%)	4 (14.8)	1 (4.5)	1.507	0.220	7 (20.0)	1 (3.4)	4.499	**0.034**
90‐day death, *N* (%)	2 (7.4)	2 (9.1)	0.046	0.831	0 (0.0)	1 (3.4)	1.602	0.206

Note

Statistical significances are indicated in bold.

DWMH: deep white matter hyperintensity; END: early neurological deterioration; FMS: Fugl‐Meyer rating scale; HT: hemorrhagic transformation; NIHSS: National Institute of Health Stroke Scale; PVH: periventricular hyperintensity; *SD*: standard deviation; sICH: symptomatic intracranial hemorrhage; TIA: transient ischemic attack.

a *t* test; others: chi‐square value.

### 3.3 Comparisons between the two subgroups in PVH patients or DWMH patients

As shown in Table 3, there were no significant differences in age, sex, risk factors for stroke, baseline NIHSS score, and baseline FMS score between the thrombolysis group and the control group in PVH patients and DWMH patients. By comparison, in either PVH patients or DWMH patients, there were no significant differences in 24‐hr NIHSS score, sICH, recurrent stroke, and 90‐day death (Table 3). Although there was no significant difference in END and HT between the thrombolysis group and the control group in PVH patients, DWMH patients undergoing IVT had a higher risk of END and HT (Table 3). Moreover, the two subgroups displayed a similar 90‐day FMS score (Table 3). Collectively, these results showed that DWMH patients and PVH patients differentially benefited from IVT.

**Table 3:** Comparison of baseline and clinical data between subgroup in patients with PVH and DWMH

Characteristics	PVH		*t/x* ^2^	*p*	DWMH		*t/x* ^2^	*p*
	Thrombolysis (*n* = 27)	Control (*n* = 35)			Thrombolysis (*n* = 22)	Control (*n* = 29)		
Age (mean ± *SD*)	68.0 ± 12.3	68.8 ± 8.9	1.603^a^	0.210	63.2 ± 12.7	68.8 ± 10.0	0.816^a^	0.371
Male, *N* (%)	18 (66.7)	16 (45.7)	2.702	0.100	16 (72.7)	17 (58.6)	1.090	0.296
Risk factors								
Hypertension, *N* (%)	18 (66.7)	26 (74.3)	1.575	0.209	20 (90.9)	27 (93.1)	0.083	0.774
Diabetes mellitus, *N* (%)	9 (33.3)	19 (54.3)	2.702	0.100	9 (40.9)	16 (55.2)	1.018	0.313
Hyperlipidemia, *N* (%)	7 (25.9)	10 (28.6)	0.054	0.817	5 (22.7)	9 (31.0)	0.433	0.510
Coronary artery disease, *N* (%)	3 (11.1)	3 (8.6)	0.112	0.738	2 (9.1)	4 (13.8)	0.272	0.602
Arterial fibrillation, *N* (%)	3 (11.1)	4 (11.4)	0.002	0.969	2 (9.1)	3 (10.3)	0.022	0.881
TIA, *N* (%)	2 (7.4)	3 (8.6)	0.028	0.867	4 (18.2)	2 (6.9)	1.528	0.216
Smoking, *N* (%)	10 (37.0)	9 (25.7)	0.919	0.338	9 (40.9)	8 (27.6)	0.999	0.318
Drinking, *N* (%)	6 (22.1)	6 (17.1)	0.252	0.616	4 (18.2)	3 (10.3)	0.642	0.423
Baseline NIHSS score (mean ± *SD*)	10.2 ± 4.0	8.8 ± 3.3	1.492^a^	0.141	9.0 ± 3.8	8.9 ± 2.8	0.026^a^	0.980
24‐hr NIHSS score (mean ± *SD*)	8.2 ± 7.0	8.8 ± 3.7	0.417^a^	0.679	7.9 ± 5.4	9.0 ± 4.0	0.886^a^	0.380
Baseline FMS score (mean ± *SD*)	83.0 ± 9.1	86.4 ± 8.3	1.512^a^	0.136	86.3 ± 8.7	85.5 ± 8.0	0.352^a^	0.726
90‐day FMS score (mean ± *SD*)	80.9 ± 26.1	84.8 ± 24.3	0.609^a^	0.545	89.7 ± 21.3	91.7 ± 7.4	0.471^a^	0.640
Clinical variables								
END, *N* (%)	3 (11.1)	5 (14.3)	0.138	0.710	8 (36.4)	3 (10.3)	5.050	**0.025**
HT, *N* (%)	1 (3.7)	3 (8.6)	0.633	0.426	5 (22.7)	1 (3.4)	4.664	**0.031**
sICH, *N* (%)	1 (3.7)	1 (2.9)	0.035	0.852	1 (4.5)	1 (3.4)	0.040	0.842
Recurrent stroke, *N* (%)	4 (14.8)	7 (20.0)	0.284	0.594	1 (4.5)	1 (3.4)	0.040	0.842
90‐day death, *N* (%)	2 (7.4)	0 (0.0)	3.412	0.065	2 (9.1)	1 (3.4)	0.716	0.398

Note

Statistical significances are indicated in bold.

DWMH: deep white matter hyperintensity; END: early neurological deterioration; FMS: Fugl‐Meyer rating scale; HT: hemorrhagic transformation; NIHSS: National Institute of Health Stroke Scale; PVH: periventricular hyperintensity; *SD*: standard deviation; sICH: symptomatic intracranial hemorrhage; TIA: transient ischemic attack.

a *t* test; others: chi‐square value.

### 3.4 Association between stroke recurrence and PVH or DWMH

**Figure 1:**
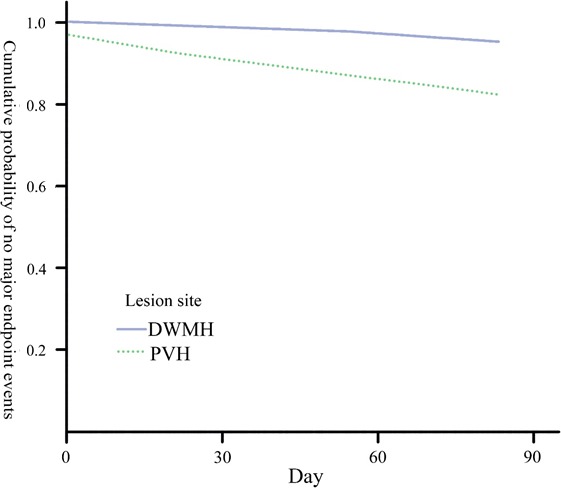
Kaplan–Meier survival curves for patients with no major endpoint events by periventricular hyperintensity (PVH) and deep white matter hyperintensity (DWMH; *p* value [log‐rank] = 0.039)

During 90‐day follow‐up, the incidence of stroke recurrence was 11.5%. Kaplan–Meier analysis revealed that there was significant difference in cumulative probability of major endpoint events between PVH patients and DWMH patients (log‐rank test [Mantel–Cox]; chi‐square = 4.252; *p* = 0.039; Figure 1). Furthermore, considering PVH, age, baseline NIHSS score, baseline FMS score, sex, hypertension, diabetes mellitus, coronary artery disease, arterial fibrillation, TIA, and thrombolytic selection, the multivariate Cox model analysis showed that PVH and TIA are two independent predictors of stroke recurrence for patients with AIS (Table 4).

**Table 4:** Multivariate Cox regression analysis on independent predictors for stroke recurrence in AIS patients

Characteristics	HR	95% CI	*p*
PVH	5.395	1.104 ~ 26.359	**0.037**
Age	1.040	0.966 ~ 1.120	0.295
Baseline NIHSS score	1.095	0.705 ~ 1.701	0.686
Baseline FMS score	1.013	0.834 ~ 1.229	0.899
Sex	1.334	0.336 ~ 5.305	0.682
Hypertension	3.558	0.387 ~ 32.746	0.262
Diabetes mellitus	1.873	0.454 ~ 7.724	0.385
Coronary artery disease	0.432	0.040 ~ 4.661	0.489
Arterial fibrillation	3.020	0.762 ~ 11.970	0.116
TIA	6.905	1.367 ~ 34.879	**0.019**
Thrombolytic selection	0.609	0.161 ~ 2.304	0.465

Note

Statistical significances are indicated in bold.

FMS: Fugl‐Meyer rating scale; NIHSS: National Institute of Health Stroke Scale; PVH: periventricular hyperintensity; TIA: transient ischemic attack.

## 4 DISCUSSION

Before this study, our previous data showed that WHM influences the therapeutic effect and prognosis of patients with cerebral infarction in the middle cerebral artery blood supply area after IVT (Supporting Information Table S1). In this study, we further showed that the efficacy of IVT is different in AIS patients with WMH at different sites. As compared to PVH patients, DWMH patients had a higher risk of END and HT after IVT. Specifically, PVH patients had a higher risk of recurrent stroke and a poorer motor function outcome relative to DWMH patients. By adjustment of confounding factors, multivariate Cox regression model revealed that PVH and TIA are two independent risk factors for recurrent stroke in AIS patients.

White matter hyperintensity, as an important type of cerebral small vessel disease, has attracted much attention recently. Although its mechanisms are unclear, increasing evidence on the association between MRI and pathology suggested that WMH at different sites might have different pathological mechanisms. Specifically, DWMH was associated with hypertension‐related atherosclerosis and severe vascular stenosis (Deicken, Reus, Manfredi, & Wolkowitz, 1991), leading to prolonged hypoperfusion of distal small arteries (Spilt et al., 2006). Moreover, the deep WM is located in the ischemic sensitive area with less collateral circulation. Eventually, deep WM reflects a chronic ischemic change. This was in line with our results showing that DWMH patients had a higher occurrence of hypertension, had a higher risk of HT, and were prone to END after IVT as compared to PVH patients. The DWM is mainly supplied by the branches of the middle cerebral artery and is susceptible to risk factors such as hypertension, consistent with our conclusion. Long‐term hypertension leads to vitreous degeneration of arterioles, thickening of the walls, and stenosis of the lumen, resulting in chronic ischemic, hypoxia of distal small arteries, further impaired vascular endothelium, increased vascular permeability, and various pathological changes in white matter (Grueter & Schulz, 2012; Uh, Yezhuvath, Cheng, & Lu, 2010; Wardlaw, Sandercock, Dennis, & Starr, 2003). Once cerebral infarction occurs in the blood supply area of the middle cerebral artery, the ischemic penumbra around the infarct cannot be saved timely, and the distal arteries at lesioned sites display prolonged hypoperfusion which further aggravates injury of the vascular wall, the infarct size will increase and the impairment of neurological functions will aggravate. Prolonged reperfusion time and a large infarct size also increase the risk of intracranial hemorrhage. In addition, increasing evidence shows that exogenous rt‐PA can further destroy the damaged blood–brain barrier, thereby affecting the hemorrhagic transformation and prognosis of patients with cerebral infarction (Ji et al., 2017; Liu et al., 2018). Previous studies showed that intracranial hemorrhage and increased infarct size are the main risk factors for the early deterioration of neurological function in patients with cerebral infarction after IVT, consistent with our findings (Seners, Turc, Oppenheim, & Baron, 2015). Together, **t**hese studies further explain the early deterioration of neurological functions and the risk of intracranial hemorrhage in cerebral infarction patients with DWMH after IVT.

In contrast, PVH has different pathological mechanisms. Plasma proteins have been found around lateral ventricles. WM lesion at these locations might not be entirely attributed to vascular factors, but likely due to the obstruction of blood circulation in the dense venous network around the ventricles, leading to higher water content in the area. Moreover, the destruction of the ependyma of lateral ventricle and the presence of glial hyperplasia of granular ependyma might lead to damage of blood–brain barrier and the leakage of cerebrospinal fluid into the periventricular region, aggravating the edema of surrounding brain tissue and nerve demyelination (Franklin & Ffrench‐Constant, 2008; Nagai, Hoshide, & Kario, 2012). Furthermore, Grafton et al. (1991) also found that PVH is associated with myelin pallor, gliosis, and Virchow–Robin space dilatation, significantly different from the pathology of DWMH. Again, Murray et al. (2012) also reported that there are more cavity formation and medulla loss in the surrounding PVH. In contrast, at the DWMH area, loss of axon, astrocyte, microglia, and oligodendrocyte was minimal. These pathological mechanisms partly at least explain a poorer recovery of motor function outcome in patients with PVH. Due to the obstruction of blood circulation in the venous network for a long time, the perivascular activity and reactivity gradually decline and self‐regulation dysfunction of cerebral microcirculation occurs, leading to the vulnerability of small vessels and further edema and infarction of surrounding brain tissue. Therefore, these are attributed to a higher risk of recurrent stroke in patients with PVH. And the latter is directly associated with the deterioration of neurological function and the poor recovery of motor function outcome at 90‐day follow‐up. Moreover, PVH was associated with the damage of corticospinal tracts, which are vital for motor function. Conceivably, PVH may be involved in the recovery of motor function in AIS patients with IVT treatment. A few of studies showed that white matter tracts located in the PVH always involve long contact fiber, whereas those located in the DWMH are more likely to involve short contact fiber (Bahrani et al., 2017; Smith et al., 2016; Spilt et al., 2006). This difference may result in the different recovery of motor function outcome.

However, there was no significant difference in 90‐day motor function outcome between PVH patients and DWMH patients after IVT. This might be related to the pathological mechanisms of WMH, including demyelination, axonal loss, oligodendrocytes, and astrocytic hyperplasia (Moon et al., 2017). It is well known that the recovery of neurological function in AIS patients depends on how the extensive neural network of the patients compensates for the damaged structures. WMH is associated with severe damage to the integrity of the brain functional network, resulting in a significant decrease in the compensatory function of the brain, and thus affecting the recovery of neurological functions (Nordahl et al., 2006). Moreover, it was reported that brain tissue perfusion was reduced by more than thirty percent in WMH patients (O'Sullivan et al., 2002), leading to poor vascular reactivity. Therefore, when AIS occurred, the reduced ischemic tolerance of brain tissue around the infarct site would result in the increase of infarct size, and eventually poor motor function outcome. Moreover, the severity of WMH might represent the overall lesion extent of small vessels, thereby affecting the prognosis after IVT. Finally, exogenous rt‐PA also increases the risk of HT, thereby increasing infarct size, aggravating the impairment of neurological function (Simao, Ustunkaya, Clermont, & Feener, 2017), and naturally affecting the recovery of motor function outcome.

Notably, limitations of our study need consideration. First, our study was a retrospective single‐center study with a small number of patients. Thus, our findings need to be confirmed by a multicenter study with a large sample size. Second, we assessed the degree of WMH and the site of WMH using MRI; thus, the subjective factor and selection bias could not completely be excluded. Third, we failed to evaluate recanalization status and cerebral microbleed burden（because only a small number of patients (12.4%) had MRI images with SWI sequence (Supporting Information Figure S2), we could not make further analysis (Tsivgoulis et al., 2016), which was associated with stroke functional outcome in previous studies (Zhang et al., 2017). Fourth, since HT occurred in a small number of patients, we failed to divide into subgroups according to the severity. Thus, mild types of HT which may be often clinically irrelevant could be not excluded. Fifth, confidence intervals in the multivariable analysis were very wide with the lower margin value close to 1 due to some uncertainties from a limited sample size.

## 5 CONCLUSION

In conclusion, this study showed that WMH at different sites might be associated with different mechanisms, which influences the efficacy of IVT and the prognosis in AIS patients. Patients with DWMH but not with PVH were associated with END and a higher risk of HT after IVT. During 90‐day follow‐up, patients with PVH but not with DWMH had a higher risk of recurrent stroke and a poorer recovery of motor function outcome. However, there was no significant difference in the recovery of motor function between patients with PVH and patients with DWMH after IVT.

## CONFLICT OF INTEREST

The authors declared no conflict of interest.

## References

[brb31149-bib-0001] Bahrani, A. A. , Powell, D. K. , Yu, G. , Johnson, E. S. , Jicha, G. A. , & Smith, C. D. (2017). White matter hyperintensity associations with cerebral blood flow in elderly subjects stratified by cerebrovascular risk. Journal of Stroke and Cerebrovascular Diseases, 26, 779–786. 28063772 10.1016/j.jstrokecerebrovasdis.2016.10.017 PMC5473621

[brb31149-bib-0002] Deicken, R. F. , Reus, V. I. , Manfredi, L. , & Wolkowitz, O. M. (1991). MRI deep white matter hyperintensity in a psychiatric population. Biological Psychiatry, 29, 918–922. 10.1016/0006-3223(91)90058-T 2049490

[brb31149-bib-0003] Erdur, H. , Scheitz, J. F. , Ebinger, M. , Rocco, A. , Grittner, U. , Meisel, A. , … Nolte, C. H. (2015). In‐hospital stroke recurrence and stroke after transient ischemic attack: Frequency and risk factors. Stroke, 46, 1031–1037. 25737318 10.1161/STROKEAHA.114.006886

[brb31149-bib-0004] Fazekas, F. , Kleinert, R. , Offenbacher, H. , Schmidt, R. , Kleinert, G. , Payer, F. , … Lechner, H. (1993). Pathologic correlates of incidental MRI white matter signal hyperintensities. Neurology, 43, 1683–1689. 10.1212/WNL.43.9.1683 8414012

[brb31149-bib-0005] Feng, C. , Tan, Y. , Wu, Y. F. , Xu, Y. , Hua, T. , Huang, J. , & Liu, X. Y. (2014). Leukoaraiosis correlates with the neurologic deterioration after small subcortical infarction. Journal of Stroke and Cerebrovascular Diseases, 23, 1513–1518. 10.1016/j.jstrokecerebrovasdis.2013.12.032 24589033

[brb31149-bib-0006] Franklin, R. J. , & Ffrench‐Constant, C. (2008). Remyelination in the CNS: From biology to therapy. Nature Reviews Neuroscience, 9, 839–855. 10.1038/nrn2480 18931697

[brb31149-bib-0007] Gao, Z. , Wang, W. , Wang, Z. , Zhao, X. , Shang, Y. , Guo, Y. , … Wu, W. (2014). Cerebral microbleeds are associated with deep white matter hyperintensities, but only in hypertensive patients. PLoS One, 9, e91637 10.1371/journal.pone.0091637 24626222 PMC3953489

[brb31149-bib-0008] Gilberti, N. , Gamba, M. , Premi, E. , Costa, A. , Vergani, V. , Delrio, I. , … Magoni, M. (2017). Leukoaraiosis is a predictor of futile recanalization in acute ischemic stroke. Journal of Neurology, 264, 448–452. 10.1007/s00415-016-8366-y 28004198

[brb31149-bib-0009] Gladstone, D. J. , Danells, C. J. , & Black, S. E. (2002). The fugl‐meyer assessment of motor recovery after stroke: A critical review of its measurement properties. Neurorehabilitation and Neural Repair, 16, 232–240. 10.1177/154596802401105171 12234086

[brb31149-bib-0010] Grafton, S. T. , Sumi, S. M. , Stimac, G. K. , Alvord, E. C. Jr , Shaw, C. M. , & Nochlin, D. (1991). Comparison of postmortem magnetic resonance imaging and neuropathologic findings in the cerebral white matter. Archives of Neurology, 48, 293–298. 1705796 10.1001/archneur.1991.00530150061019

[brb31149-bib-0011] Grueter, B. E. , & Schulz, U. G. (2012). Age‐related cerebral white matter disease (leukoaraiosis): A review. Postgraduate Medical Journal, 88, 79–87. 10.1136/postgradmedj-2011-130307 22184252

[brb31149-bib-0012] Jeong, H. G. , Kim, B. J. , Yang, M. H. , Han, M. K. , & Bae, H. J. (2015). Neuroimaging markers for early neurologic deterioration in single small subcortical infarction. Stroke, 46, 687–691. 25677600 10.1161/STROKEAHA.114.007466

[brb31149-bib-0013] Ji, B. , Zhou, F. , Han, L. , Yang, J. , Fan, H. , Li, S. , … Xu, Y. (2017). Sodium tanshinone IIA sulfonate enhances effectiveness Rt‐PA treatment in acute ischemic stroke patients associated with ameliorating blood‐brain barrier damage. Translational Stroke Research, 8, 334–340. 28243834 10.1007/s12975-017-0526-6 PMC5493726

[brb31149-bib-0014] Johnston, K. C. , Connors, A. F. Jr , Wagner, D. P. , Knaus, W. A. , Wang, X. , & Haley, E. C. Jr (2000). A predictive risk model for outcomes of ischemic stroke. Stroke, 31, 448–455. 10657421 10.1161/01.str.31.2.448

[brb31149-bib-0015] Kimura, K. , Iguchi, Y. , Shibazaki, K. , Iwanaga, T. , Yamashita, S. , & Aoki, J. IV (2009). t‐PA therapy in acute stroke patients with atrial fibrillation. Journal of the Neurological Sciences, 276, 6–8. 19010485 10.1016/j.jns.2008.10.018

[brb31149-bib-0016] Kongbunkiat, K. , Wilson, D. , Kasemsap, N. , Tiamkao, S. , Jichi, F. , Palumbo, V. , … Werring, D. J. (2017). Leukoaraiosis, intracerebral hemorrhage, and functional outcome after acute stroke thrombolysis. Neurology, 88, 638–645. 10.1212/WNL.0000000000003605 28130468 PMC5317383

[brb31149-bib-0017] Larrue, V. , von Kummer, R. R. , Muller, A. , & Bluhmki, E. (2001). Risk factors for severe hemorrhagic transformation in ischemic stroke patients treated with recombinant tissue plasminogen activator: A secondary analysis of the European‐Australasian Acute Stroke Study (ECASS II). Stroke, 32, 438–441. 11157179 10.1161/01.str.32.2.438

[brb31149-bib-0018] Liu, Y. , Zhang, M. , Chen, Y. , Gao, P. , Yun, W. , & Zhou, X. (2018). The degree of leukoaraiosis predicts clinical outcomes and prognosis in patients with middle cerebral artery occlusion after intravenous thrombolysis. Brain Research, 1681, 28–33. 10.1016/j.brainres.2017.12.033 29288062

[brb31149-bib-0019] Moon, S. Y. , de Souto Barreto, P. , Chupin, M. , Mangin, J. F. , Bouyahia, A. , Fillon, L. , … Vellas, B. (2017). Associations between white matter hyperintensities and cognitive decline over three years in non‐dementia older adults with memory complaints. Journal of the Neurological Sciences, 379, 266–270. 28716257 10.1016/j.jns.2017.06.031

[brb31149-bib-0020] Murray, M. E. , Vemuri, P. , Preboske, G. M. , Murphy, M. C. , Schweitzer, K. J. , Parisi, J. E. , … Dickson, D. W. (2012). A quantitative postmortem MRI design sensitive to white matter hyperintensity differences and their relationship with underlying pathology. Journal of Neuropathology and Experimental Neurology, 71, 1113–1122. 23147507 10.1097/NEN.0b013e318277387e PMC3511604

[brb31149-bib-0021] Nagai, M. , Hoshide, S. , & Kario, K. (2012). Association of prothrombotic status with markers of cerebral small vessel disease in elderly hypertensive patients. American Journal of Hypertension, 25, 1088–1094. 10.1038/ajh.2012.85 22739806

[brb31149-bib-0022] Nordahl, C. W. , Ranganath, C. , Yonelinas, A. P. , Decarli, C. , Fletcher, E. , & Jagust, W. J. (2006). White matter changes compromise prefrontal cortex function in healthy elderly individuals. Journal of Cognitive Neuroscience, 18, 418–429. 10.1162/jocn.2006.18.3.418 16513006 PMC3776596

[brb31149-bib-0023] O'Sullivan, M. , Lythgoe, D. J. , Pereira, A. C. , Summers, P. E. , Jarosz, J. M. , Williams, S. C. , & Markus, H. S. (2002). Patterns of cerebral blood flow reduction in patients with ischemic leukoaraiosis. Neurology, 59, 321–326. 12177363 10.1212/wnl.59.3.321

[brb31149-bib-0024] Pantoni, L. (2010). Cerebral small vessel disease: From pathogenesis and clinical characteristics to therapeutic challenges. The Lancet Neurology, 9, 689–701. 10.1016/S1474-4422(10)70104-6 20610345

[brb31149-bib-0025] Parsons, M. W. , Barber, P. A. , Desmond, P. M. , Baird, T. A. , Darby, D. G. , Byrnes, G. , … Davis, S. M. (2002). Acute hyperglycemia adversely affects stroke outcome: A magnetic resonance imaging and spectroscopy study. Annals of Neurology, 52, 20–28. 12112043 10.1002/ana.10241

[brb31149-bib-0026] Seners, P. , Turc, G. , Oppenheim, C. , & Baron, J. C. (2015). Incidence, causes and predictors of neurological deterioration occurring within 24 h following acute ischaemic stroke: A systematic review with pathophysiological implications. Journal of Neurology, Neurosurgery and Psychiatry, 86, 87–94. 10.1136/jnnp-2014-308327 24970907

[brb31149-bib-0027] Shim, Y. S. , Yang, D. W. , Roe, C. M. , Coats, M. A. , Benzinger, T. L. , Xiong, C. , … Morris, J. C. (2015). Pathological correlates of white matter hyperintensities on magnetic resonance imaging. Dementia and Geriatric Cognitive Disorders, 39, 92–104. 10.1159/000366411 25401390 PMC4312498

[brb31149-bib-0028] Shrestha, I. , Takahashi, T. , Nomura, E. , Ohtsuki, T. , Ohshita, T. , Ueno, H. , … Matsumoto, M. (2009). Association between central systolic blood pressure, white matter lesions in cerebral MRI and carotid atherosclerosis. Hypertension Research, 32, 869–874. 19644503 10.1038/hr.2009.121

[brb31149-bib-0029] Simao, F. , Ustunkaya, T. , Clermont, A. C. , & Feener, E. P. (2017). Plasma kallikrein mediates brain hemorrhage and edema caused by tissue plasminogen activator therapy in mice after stroke. Blood, 129, 2280–2290. 28130211 10.1182/blood-2016-09-740670 PMC5399481

[brb31149-bib-0030] Smith, C. D. , Johnson, E. S. , Van Eldik, L. J. , Jicha, G. A. , Schmitt, F. A. , Nelson, P. T. , … Wellnitz, C. V. (2016). Peripheral (deep) but not periventricular MRI white matter hyperintensities are increased in clinical vascular dementia compared to Alzheimer's disease. Brain Behav, 6, e00438 10.1002/brb3.438 26925303 PMC4754499

[brb31149-bib-0031] Spilt, A. , Goekoop, R. , Westendorp, R. G. , Blauw, G. J. , de Craen, A. J. , & van Buchem, M. A. (2006). Not all age‐related white matter hyperintensities are the same: A magnetization transfer imaging study. AJNR. American Journal of Neuroradiology, 27, 1964–1968. 17032876 PMC7977888

[brb31149-bib-0032] Tsivgoulis, G. , Zand, R. , Katsanos, A. H. , Turc, G. , Nolte, C. H. , Jung, S. , … Alexandrov, A. V. (2016). Risk of symptomatic intracerebral hemorrhage after intravenous thrombolysis in patients with acute ischemic stroke and high cerebral microbleed burden: A meta‐analysis. JAMA Neurology, 73, 675–683. 10.1001/jamaneurol.2016.0292 27088650

[brb31149-bib-0033] Uh, J. , Yezhuvath, U. , Cheng, Y. , & Lu, H. (2010). In vivo vascular hallmarks of diffuse leukoaraiosis. Journal of Magnetic Resonance Imaging, 32, 184–190. 10.1002/jmri.22209 20578025 PMC3236451

[brb31149-bib-0034] Wardlaw, J. M. , Sandercock, P. A. , Dennis, M. S. , & Starr, J. (2003). Is breakdown of the blood‐brain barrier responsible for lacunar stroke, leukoaraiosis, and dementia? Stroke, 34, 806–812. 10.1161/01.STR.0000058480.77236.B3 12624314

[brb31149-bib-0035] Wardlaw, J. M. , Smith, C. , & Dichgans, M. (2013). Mechanisms of sporadic cerebral small vessel disease: Insights from neuroimaging. The Lancet Neurology, 12, 483–497. 10.1016/S1474-4422(13)70060-7 23602162 PMC3836247

[brb31149-bib-0036] Zhang, J. H. , Obenaus, A. , Liebeskind, D. S. , Tang, J. , Hartman, R. , & Pearce, W. J. (2017). Recanalization, reperfusion, and recirculation in stroke. Journal of Cerebral Blood Flow & Metabolism, 37(12), 3818–3823. 10.1177/0271678X17732695 28925323 PMC5718333

[brb31149-bib-0037] Zhang, M. , Zhu, W. , Yun, W. , Wang, Q. , Cheng, M. , Zhang, Z. , … Xu, G. (2015). Correlation of matrix metalloproteinase‐2 single nucleotide polymorphisms with the risk of small vessel disease (SVD). Journal of the Neurological Sciences, 356, 61–64. 10.1016/j.jns.2015.04.056 26152827

[brb31149-bib-0038] Zhong, G. , Yan, S. , Zhang, S. , Chen, Q. , Lai, Y. , & Lou, M. (2016). Association between leukoaraiosis and poor outcome is not due to reperfusion inefficiency after intravenous thrombolysis. Translational Stroke Research, 7, 439–445. 10.1007/s12975-016-0473-7 27256491

