# Supplementary material for: The efficacy of intravenous thrombolysis in acute ischemic stroke patients with white matter hyperintensity
Source: Brain Behav. 2018 Oct 31;8(12):e01149. doi: 10.1002/brb3.1149 (PMC6305931; doi:10.1002/brb3.1149)
Supplement: Supplementary file 1 [file BRB3-8-e01149-s001.docx]

Table S1. The comparisons of baseline characteristics and clinical information between WHM group and control group after IVT

| Characteristics | WHM group  (n=24) | Control group  (n=54) | | *t/* *x*^2^ | | *P* |
| --- | --- | --- | --- | --- | --- | --- |
| Age (years, mean ± SD) | 74±7 | 61±11 | 6.567^a^ | | 0.012 | |
| Male, N (%) | 14(58.3) | 40(74.1) | 1.933 | | 0.164 | |
| Hypertension, N (%) | 19(79.2) | 38(70.4) | 0.653 | | 0.419 | |
| Diabetes mellitus, N (%) | 11(45.8) | 16(29.6) | 1.927 | | 0.165 | |
| Coronary artery disease, N (%) | 3(12.5) | 8(14.8) | 0.075 | | 0.784 | |
| Arterial fibrillation, N (%) | 5(20.8) | 10(22.2) | 0.057 | | 0.812 | |
| Smoking, N (%) | 8(33.3) | 29(53.7) | 2.765 | | 0.096 | |
| Drinking, N (%) | 5(20.8) | 20(37.0) | 2.003 | | 0.157 | |
| Baseline NIHSS score (mean ± SD) | 12±6 | 11±5 | 1.981^a^ | | 0.163 | |
| Time to thrombolysis |  |  | 0.630 | | 0.427 | |
| ≤3.0h | 11(45.8) | 30(55.6) |  | |  | |
| ＞3.0~4.5h | 13(54.2) | 24(44.4) |  | |  | |
| Clinical variables |  |  |  | |  | |
| HT, N (%) | 8(33.3) | 6(11.1) | 5.571 | | **0.018** | |
| sICH, N (%) | 4(16.7) | 3(5.6) | 2.304 | | 0.129 | |
| Initial FMS score (mean ± SD) | 70.4±3.8 | 77±3 | 2.736^a^ | | 0.102 | |
| 90d FMS score (mean ± SD) | 73±8 | 92±3 | 22.345^a^ | | **<0.001** | |
| Recurrence of stroke, N (%) | 5(20.8) | 3(5.6) | 3.850 | | **0.050** | |
| 90d death rate | 5(20.5) | 1(0.02) | 5.970 | | **0.015** | |

WMH: deep white matter hyperintensity; NIHSS: National Institute of Health Stroke Scale; FMS: Fugl-Meyer rating scale; HT: hemorrhagic transformation; sICH: symptomatic intracranial hemorrhage; SD: standard deviation. Statistical significances are indicated in bold.

**Figure S1**

**
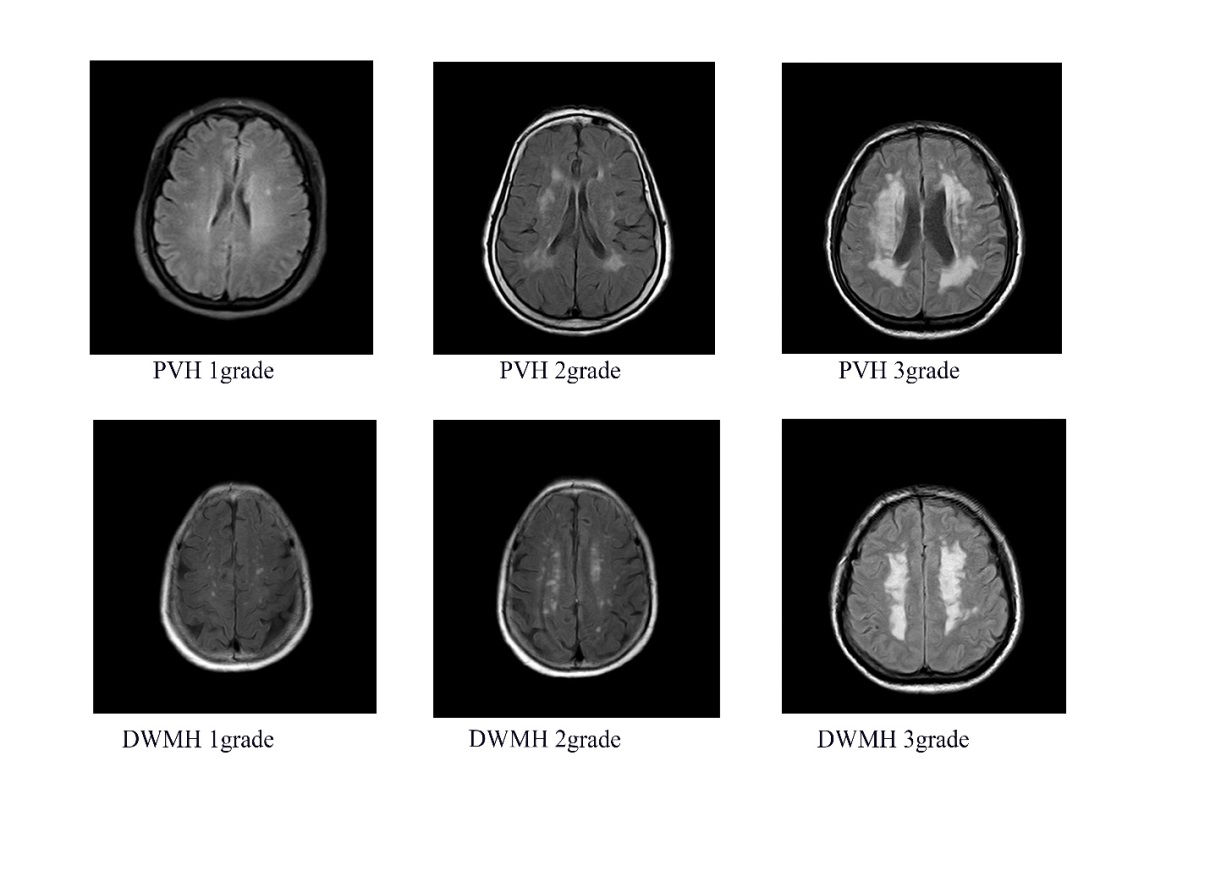
**

Figure S1. Representative images for PVH1-3 grade and DWMH 1-3 grade. PVH:

periventricular hyperintensity; DWMH: deep white matter hyperintensity.

**Figure S2**


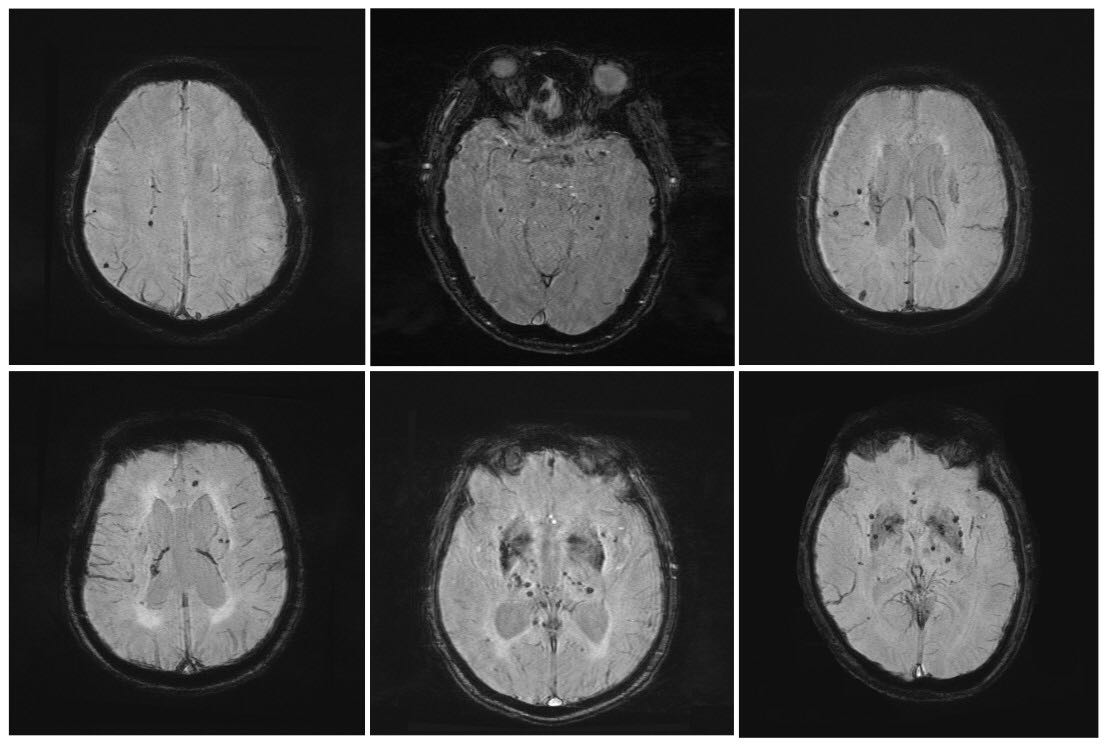


Figure S2 Representative images (SWI sequence) from six patients.
